# Controlling Gas Generation of Li-Ion Battery through Divinyl Sulfone Electrolyte Additive

**DOI:** 10.3390/ijms23137328

**Published:** 2022-06-30

**Authors:** Woon Ih Choi, Insun Park, Jae Sik An, Dong Young Kim, Meiten Koh, Inkook Jang, Dae Sin Kim, Yoon-Sok Kang, Youngseon Shim

**Affiliations:** 1 Innovation Center, Samsung Electronics, 1 Samsungjeonja-ro, Hwasung 18448, Korea; wooni.choi@samsung.com (W.I.C.); jaesik.an@samsung.com (J.S.A.); inkook.jang@samsung.com (I.J.); daesin.kim@samsung.com (D.S.K.); 2 Samsung Advanced Institute of Technology (SAIT), Samsung Electronics, 130 Samsung-ro, Suwon 16678, Korea; insun01.park@samsung.com (I.P.); dy777.kim@samsung.com (D.Y.K.); mc.koh@samsung.com (M.K.)

**Keywords:** lithium-ion battery, liquid electrolyte, additive, electrochemical reaction, gas generation, density functional theory, ab initio molecular dynamics

## Abstract

The focus of mainstream lithium-ion battery (LIB) research is on increasing the battery’s capacity and performance; however, more effort should be invested in LIB safety for widespread use. One aspect of major concern for LIB cells is the gas generation phenomenon. Following conventional battery engineering practices with electrolyte additives, we examined the potential usage of electrolyte additives to address this specific issue and found a feasible candidate in divinyl sulfone (DVSF). We manufactured four identical battery cells and employed an electrolyte mixture with four different DVSF concentrations (0%, 0.5%, 1.0%, and 2.0%). By measuring the generated gas volume from each battery cell, we demonstrated the potential of DVSF additives as an effective approach for reducing the gas generation in LIB cells. We found that a DVSF concentration of only 1% was necessary to reduce the gas generation by approximately 50% while simultaneously experiencing a negligible impact on the cycle life. To better understand this effect on a molecular level, we examined possible electrochemical reactions through ab initio molecular dynamics (AIMD) based on the density functional theory (DFT). From the electrolyte mixture’s exposure to either an electrochemically reductive or an oxidative environment, we determined the reaction pathways for the generation of CO_2_ gas and the mechanism by which DVSF additives effectively blocked the gas’s generation. The key reaction was merging DVSF with cyclic carbonates, such as FEC. Therefore, we concluded that DVSF additives could offer a relatively simplistic and effective approach for controlling the gas generation in lithium-ion batteries.

## 1. Introduction

For the extensive use of lithium-ion batteries in portable electronic devices and vehicles, Li-ion batteries require the following characteristics: (i) high capacity, (ii) long cycle life, and (iii) affordable cost [1,2]. To achieve this goal, engineers must address the gas generation phenomenon that plagues Li-ion batteries, particularly battery cells that employ liquid electrolytes. The gas generation phenomenon is commonly associated with irreversible parasitic reactions that lead to capacity loss and degradation of battery performance [3]. Significant volumes of gas are generated in the formation cycle since solid electrolyte interphase (SEI) formation involves electrochemical reactions that produce unwanted gaseous products [4,5,6], and gaseous products may also generate spontaneously during storage. The gas generation phenomenon occurs predominantly at the interface between the electrode and electrolyte, and hence, there are two distinct approaches for stabilizing the electrode surface: (i) electrode surface treatment, and (ii) electrolyte additive addition. In the first approach, the surface treatment creates a coating layer on the electrode and stabilizes the reactive regions on the electrode surface [7]. In the latter approach, additives in the electrolyte react with the electrode surface and produce an interphase layer. While both aforementioned methods have similar aims, the use of additives can be a more cost-effective approach when an ideal additive is available. Since additives usually undergo electrochemical oxidation or reduction prior to other reactants (e.g., solvent molecules and salt anions), there are efforts to use additives to block gas generation reaction pathways [8].

In searching for an ideal additive material, our efforts focused on sulfone-based solvents, as these solvents have wide electrochemical windows with high anodic stability at high voltages [9,10,11,12,13]. In particular, fluorinated sulfone electrolytes assist in the SEI layer formation on the anode and demonstrate exceptional anodic stability in high voltage cells [9,10]. In this study, divinyl sulfone (DVSF) was the most promising additive due to the 50% reduction in gas generation for battery cells that used electrolyte mixtures with DVSF.

Ab initio molecular dynamics (AIMD) simulations were proven to be useful for electrolyte reactions [14,15] as well as for the modeling of a battery’s electrode [16,17,18]. In our study, AIMD simulations of electrolyte mixtures were performed to better understand the effects of DVSF additives. The overall model workflow was identical to the one applied in our previous study on electro-reduction reactions of an electrolyte mixture [19]. A key element of the workflow was adding a certain number (*n*) of Li^+^ excess first to the electrolyte mixture while maintaining the simulation cell charge as *n*^+^ and, at a certain point in time, supplying the same number of electrons to initiate reductive reactions. In our previous study, gaseous products predicted by the model were in fairly good agreement with the recent in situ gas measurements in literature [20]. Therefore, we applied the same workflow in this study to determine the possible mechanisms behind the effects of DVSF additives.

## 2. Results and Discussion

**Figure 1:**
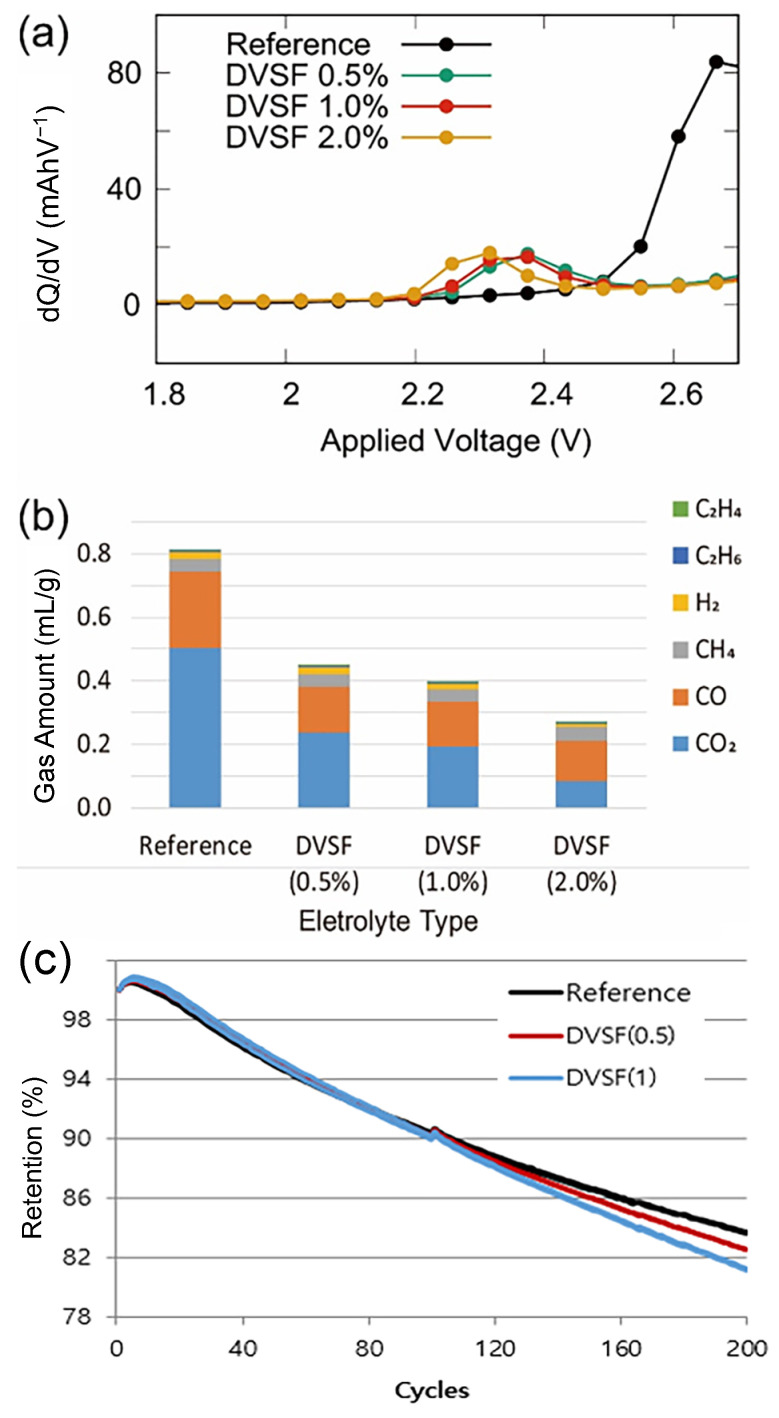
(**a**) Differential capacitance plot with respect to the applied voltage. The peak positions changed when the different amounts of DVSF were added to the reference electrolyte. (**b**) The amount of gaseous products measured with respect to the volume % of DVSF additives. (**c**) Capacity change over repeated charge-discharge (1C/1C) cycles. Up to the initial 90 cycles, the cell with 1% DVSF in the electrolyte had a slightly higher capacity than the reference cell. However, in the extended cycles, the battery cell with higher DVSF concentration had lower capacity than the reference battery cell. Note that the reference electrolyte was 1.15M of LiPF_6_ solvated in the mixture of FEC, EC, DMC, and EMC with a 5:20:35:40 volume ratio.

For a range of DVSF concentrations, the differential charge capacity (dQ/dV) as a function of the applied voltage is shown in Figure 1a. While the fluoroethylene carbonate (FEC [21]) reduction’s peak position was at ~2.65 V, the addition of DVSF shifted the peak position to ~2.3 V, and the peak height was significantly suppressed. This indicated that the LUMO energy level of DVSF was lower than the FEC solvent molecules, which was attributed to the C=C double bond in the vinyl group of DVSF.

In Figure 1b, we present the volumetric measurement data of the gaseous products generated from the battery cells. For these measurements, the fully charged battery cells (18650 type) were stored at relatively high temperatures (~60 °C) for ten consecutive days. The gas measurements could only be performed in a destructive manner to detect the various gaseous products. Regardless of the electrolyte mixture composition, the CO_2_ and CO gases account for a large fraction of the gas product volume. The most generated gas species for the reference electrolyte mixture was CO_2_; however, the relative ratio between CO_2_ and CO changed with increasing DVSF concentrations. At a DVSF content of 2%, CO became the dominant product species, which implied that the DVSF additive prohibited the reaction pathway of CO_2_ generation more effectively than CO did. The total volume of gas generation became less than 40% when 2wt% of the DVSF additive was mixed with the reference electrolyte. The CO_2_ gas that evolved in the lithium-ion battery could have originated from several candidates, such as cathode materials, surface free lithium compounds (i.e., Li_2_CO_3_ and LiOH) [22,23,24], the main solvent in the electrolyte (carbonates) [20,25], or the most likely candidate, fluoroethylene carbonate (FEC).

In contrast to the reduction of CO_2_ gas generation by DVSF, the CO gas generation did not decrease further as the concentration of DVSF increased from 0.5% to 2.0%. A fraction of CO gas was generated from other cyclic carbonates (e.g., EC) [26,27]; however, blocking its reaction pathway was not as effective as the CO_2_ gas generation’s prevention by FEC. This phenomenon is discussed further in the presentation of the computational modeling results.

Despite the effective reduction of gas generation, one may question whether there are any negative side effects on the battery’s performance. Therefore, as shown in Figure 1c, we investigated the change in the capacity as a function of the number of charge-discharge cycles. Compared with the reference cell, a negligible difference was found for the initial 90 cycles, and noticeable capacity loss was found for >90 charge-discharge cycles. After 200 cycles, the battery cell’s capacity with 1% of DVSF in the electrolyte was 2.4% less than the reference cell. This degree of capacity loss was in the acceptable range [28,29,30]; however, a further loss could be expected if more DVSF is added to the electrolyte, and additional care is needed when considering the application of higher DVSF concentrations [31,32].

In order to examine the effect of FEC, battery cells with two different electrolyte mixtures were prepared: (i) EC:DMC:EMC with a ratio of 20:40:40, and (ii) FEC:EC:DMC:EMC with a ratio of 5:20:35:40. When we compared the generated gas species after ten consecutive days of storage at 60 °C, we found that the CO_2_ generated from the cell that used the electrolyte with 5% of FEC stood out. Therefore, we can conclude that CO_2_ generation is closely associated with the electrochemical reactions of FEC molecules, and DVSF additives may disrupt FEC reaction pathways.

In this study, a destructive approach was used in the gas measurements; hence, continuous monitoring of the gas generation in a single battery cell was impossible. Therefore, several identical battery cells were manufactured in order to overcome this obstacle. By measuring the gas generation of those battery cells at different stages, we could indirectly trace the volume of each gaseous species as a function of time. For this study, three battery cells were prepared to measure the gas in the following three stages: (i) pre-charged to 60% of battery capacity at a rate of 0.2C and then stored for 24 h at 25 °C; (ii) 10 consecutive days of storage at 60 °C; and (iii) 30 consecutive days of storage at 60 °C.

**Figure 2:**
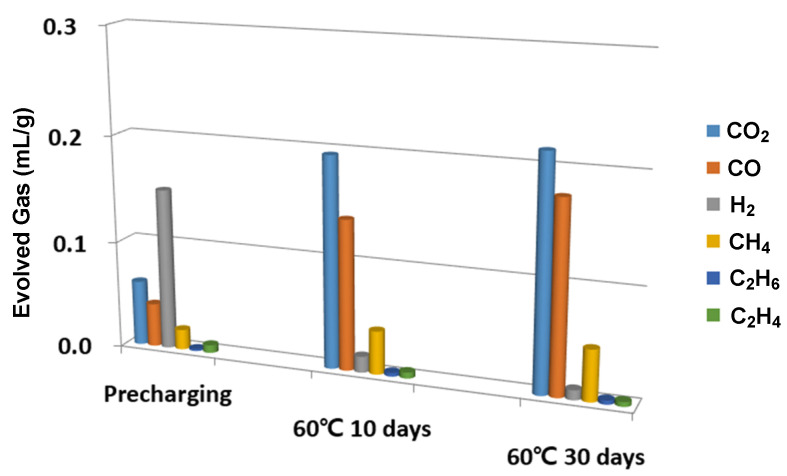
In order to show how the relative ratio of gaseous species changed over time, we showed the gas analysis results, particularly for the three cells that belong to three different stages. The pre-charging cell was charged up to 60% of capacity with a 0.2C rate and stored for one day at 25 °C. For the other two cells, we stored them for 10 and 30 days after fully changing them.

As shown in Figure 2, the composition of the generated gas changed over time. The significant amount of hydrogen generated after pre-charging became much smaller after the ten days of storage at 60 °C. We speculated that other reactions consumed the H2 molecules while the cell was stored. On the other hand, the gas measurement data show that the CO2 and CO volumes continuously increased and became the dominant gas species after 10 and 30 days of storage. If gaseous species are generated mainly during pre-charging or the formation cycle, the appropriate modeling must include reductive reactions of the electrolyte mixture. However, our experiments demonstrated that gas species were generated during spontaneous discharging when electrolytes were exposed to an electro-oxidative environment. Therefore, additional AIMD was performed in an electro-oxidative environment after the electrolyte mixture underwent initial electro-reductive reactions.

Since the only cyclic carbonate we considered in the modeling was FEC, the gaseous products found in AIMD were predominantly CO and CO_2_. The numbers of gas molecules from the electrolyte with and without DVSF additives are listed in Table 1. Please note that we sampled ten different structures for a single electrolyte composition; however, the solvation structures varied. Therefore, the generated gas species differed when each sampled structure went through the reduction and oxidation cycle. This indicated that the solvation structure at the moment when electrons were supplied played a critical role in electrochemical reactions, including gas generation. For the quantitative evaluation, we used the simple summation of the total number of gas molecules from 10 different structures. In electrolytes without DVSF, 11 CO molecules and 0 CO_2_ were generated after the electro-reductive reaction, as shown in Table 1. After oxidation, 14 CO and 7 CO_2_ molecules were generated. Noticeably, oxidation produced more CO_2_ molecules. This result was consistent with the previous study in that the CO_2_ molecule generation from reductive reactions is rare. Furthermore, this also implied that CO_2_ generation might be associated with secondary oxidation reactions of the reduced electrolyte. The number of generated gas molecules for the electrolyte mixture with DVSF was much fewer. Only one and two CO_2_ molecules were generated in the reduction and oxidation reactions, respectively. Note that the DVSF concentration (20% in number) was more than that of electrolyte (a few % in volume) in real experiments. Therefore, its influence on the gas amount became significant (21 vs. 2) compared with the experiment.

**Figure 3:**
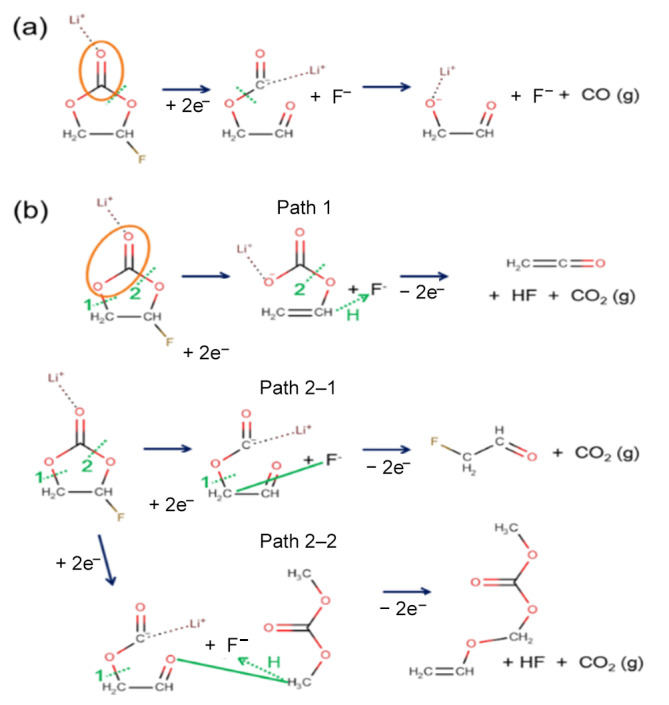
Reaction mechanism of (**a**) CO and (**b**) CO_2_ gas generation out of FEC. CO gas molecules are generated mainly from electro-reductive reactions. However, CO_2_ molecules are generated when reduced FEC goes through oxidative reactions. The dotted green line indicates the bond breaking, and the orange oval is the moiety that broke off to become gas molecules after the reaction. In addition, the solid green lines indicate the formation of the new bond. The dotted green line with an arrow shows the transfer of hydrogen atom.

The typical reaction pathways for CO and CO_2_ gas generation that were observed in AIMD modeling are summarized in Figure 3a,b, respectively. While CO generation was frequently found in the reductive reaction [33], an additional oxidation reaction was necessary to observe CO_2_ generation. The initial ring-opening and F-abstraction occurred near-simultaneously to create two atomic sites for additional electron accommodation (C^−^ and F^−^). An electron from a carbon atom was transferred to a neighboring oxygen atom (O^−^), which led to the generation of a CO molecule. For CO_2_ generation, two different reaction pathways could occur, depending on the C–O bond’s breaking sequence, as shown in Figure 3b. Upon electro-reduction, similar to the CO generation in Figure 3a, the ring-opening and F-abstraction happened first (see Figure 3b). If an oxidation condition were imposed on the reduced electrolyte where the reaction had not yet proceeded to the point of CO gas generation, CO_2_ gas could be generated. Even though the details differed, the common reaction scheme for CO_2_ generation out of FEC was similar. An initial electro-reduction led to the ring-opening of the FEC molecule, which left a ring-opened carbonate with a negative charge and F^−^ ion behind. When an electro-oxidation condition was imposed in the following AIMD, the negative charge at the F and O atomic sites needed to disappear. The AIMD solution led to the formation of new bonds with neighboring atoms, and hence, the F atom formed a bond with either carbon or hydrogen. Naturally, this led to the C–O bond breaking and subsequent generation of a neutral CO_2_ molecule. Interestingly, path 2–2 produced another neutral molecule by merging the two ionic species, which had a higher molecular weight than that of the solvent molecules.

**Figure 4:**
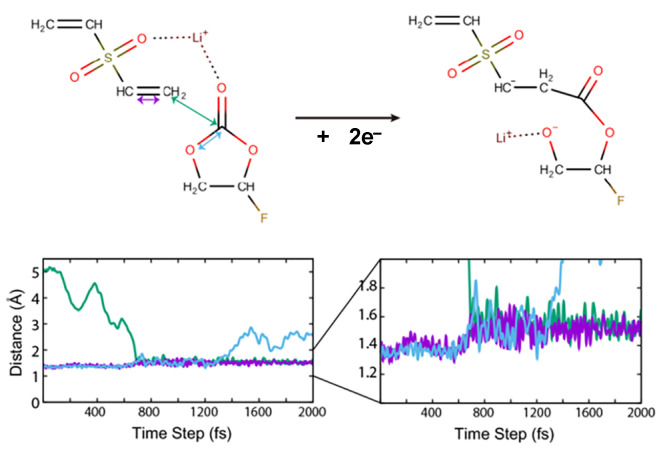
Merging of FEC with DVSF in the electro-reductive condition. One of the double-bonded carbons in vinyl moiety formed a new bond with a carbonyl carbon of FEC. The distances between characteristic atoms show the formation and breaking of bonds. The purple line indicates the distance between two carbon atoms in the vinyl moiety. The increase after the reaction indicates the decrease of bond order. The dark green line indicates C–C bond formation that leads to the merging of two molecules. The ring-opening of FEC is shown with C–O distance that is marked with cyan color.

The presence of DVSF changes the reaction pathway of the electro-reductive and the following oxidative reactions. The typical reductive reaction we found was the merging of DVSF with other cyclic carbonates such as FEC, as shown in Figure 4. As confirmed by the dQ/dV curve, the LUMO of DVSF was relatively lower than that of the other solvent molecules, such as FEC and DMC. This was attributed to the C=C anti-bonding in the vinyl group, since that was a π* character. Since the electronic occupation in the LUMO of DVSF increases the total energy of the electrolyte, the electrolyte system may alleviate this instability by forming new C–C bonds between DVSF and FEC. The change in distance between the relevant atoms offers information on the temporal sequence of new bond formation and existing bond separation. As shown in Figure 4, the ring-opening reaction occurred approximately 700 fs after the bond formation. In addition to this bond breaking and formation, the change in bond order can also be observed. Before the merging reaction, the C–C distance of vinyl carbon, which was originally in the range of the C=C double bond, became elongated to the length of a single C–C bond after the formation of the intermolecular C–C bond. As a result, a new organic ion with a negative charge was generated, and we saw that the gas generation reactions from FEC were blocked. Our simulations showed that the bond breaking from electrochemical reactions mainly occurred at the C–O bonds. As a result of the bond breaking, new atomic sites that accommodate electrons were generated. However, none of the C–O bond breaking in the structure in Figure 4 could generate gaseous molecules.

Due to the limitation on the boundary size of the model, further reactions of the ionic species generated out of the merging reaction are not available in this study. However, we noted that there was one more intact vinyl moiety even after the first merging reaction. We expect that another merging reaction will be available when additional electrons are transferred.

We deduced that this merging reaction of DVSF had a preference. As shown in the merging reaction mechanism, it was initiated by charged solvent molecules. If there are different types of solvent molecules with distinct LUMO levels, the molecules with lower LUMO levels are more likely to merge with DVSF [34,35]. In the experiments, both FEC and EC solvent molecules were present in the mixed electrolyte, and since the FEC molecules had lower LUMO levels than those of the EC molecules [35,36], merging reactions with DVSF are more likely to occur with FEC. Therefore, blocking of the gas generation reactions by EC solvent molecules was not as effective as that of FEC, which may be the reason for the lack of further CO gas generation reduction when DVSF concentrations increased from 0.5% to 2.0% (see Figure 1b). Unfortunately, experimental support for this merging reaction is not yet available since products from this merging reaction undergo additional complex reactions to eventually become a certain type of interphase layer (SEI, solid electrolyte interphase) on the electrode surface.

**Figure 5:**
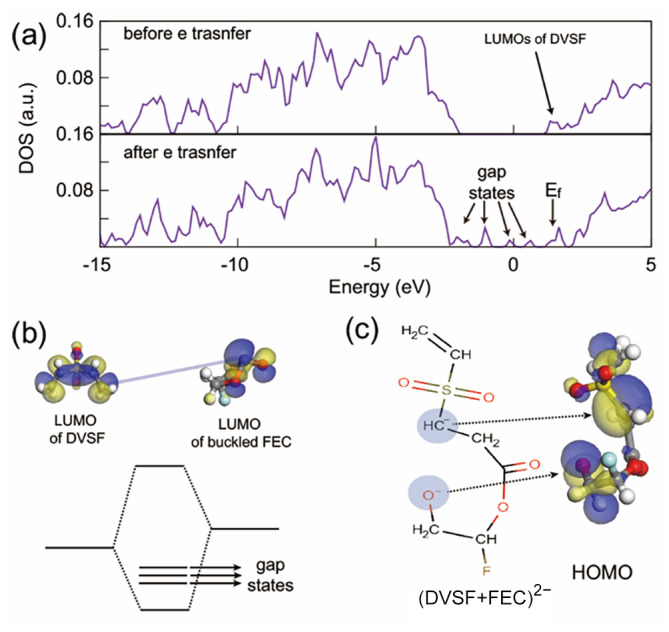
(**a**) Total density of states (TDOS) of the electrolyte mixture. The LUMO of divinyl sulfone (DVSF) was also the LUMO of the whole electrolyte system; therefore, additional electrons occupied this state. (**b**) There was an interaction between the partially occupied LUMO of DVSF with the unoccupied LUMO level of buckled FEC. Therefore, this interaction resulted in a C–C bond that, in turn, formed radicals with higher molecular weights. (**c**) The electronic wave function of the gap state confirmed that the electrons were indeed located at the positions of the radicals that were marked in the molecular graph.

Electrochemical reactions are basically driven by electrons. Therefore, to better understand chemical bond rearrangements such as bond formation and breaking, it is necessary to study the change in electronic structures. As shown in Figure 5a, the total density of states (TDOS) of the electrolyte mixture showed peaks relevant to the LUMO of DVSF. Before adding additional electrons, five LUMO levels of DVSF are distributed right below the LUMO levels of FEC. From the nodal planes of the LUMO wave function, it was easily recognized that the LUMO level had a π* character. If electrons were added to this system, the electrons would occupy one of the LUMO of DVSF and, at the same time, the LUMO level of a nearby FEC molecule. With this additional electron, the carbonyl carbon of FEC moved out of the CO_3_ plane. As shown in Figure 5b, the LUMO wave function of buckled FEC had a higher electronic density than the carbonyl carbon. Since the two LUMO levels were relatively high in energy, the system tried to minimize the total energy by forming a bond between the vinyl carbon and the carbonyl carbon. This significant amount of energy gain was the driving force of the merging reaction between FEC and DVSF. After the electron transfer, newly generated gap states were found in the total density of states, which indicated the bond rearrangement after the electro-reductive reactions. Among them, the occupied levels indicated atomic sites that accommodated electrons. This is shown in the plot of the wave function (see Figure 5c), where atomic sites that were expected to have electrons actually had higher electron density.

**Figure 6:**
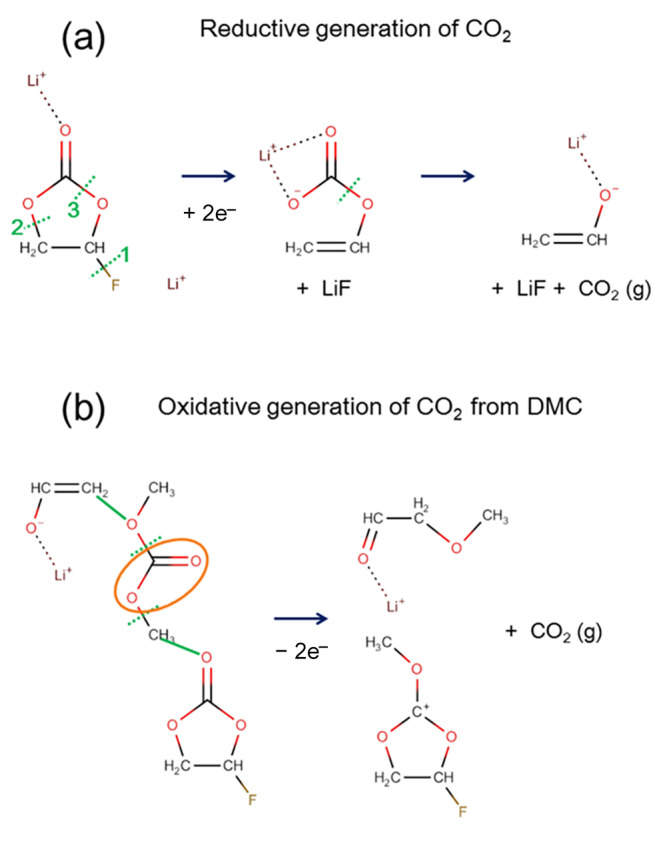
Reactions of CO_2_ gas generation were observed through AIMD modeling. Both the (**a**) reductive reaction and (**b**) oxidative reaction produced molecular CO_2_. Overall, the CO_2_ generation reactions were observed much more frequently in the electrolyte without the DVSF additive. The majority of CO_2_ gases were generated in the following oxidation rather than in the initial reductive process. The solid green lines indicate bond formation, and the dotted green lines indicate bond breaking. The atoms in the orange circle become a CO_2_ gas molecule after an oxidative reaction.

Since AIMD simulations also show gas generation, detailed reaction pathways can be analyzed. As shown in Table 1, most CO_2_ molecules were generated through the following oxidation rather than through the initial electro-reductive reactions. The relative ratio was 7:0 and 2:1 in the electrolyte without and with DVSF additive molecules, respectively. The schematics of the detailed reactions that we observed through AIMD simulations are presented in Figure 6. The solid green lines indicate bond formation, and the green dotted lines indicate bond breaking. The numbers marked in green show the sequential order of bond breaking. Upon reductive stress, the responses were mainly bond breaking, which led to the creation of atomic sites that could accommodate additional electrons. However, upon oxidative stress, bond breaking and bond formation took place simultaneously. Since electrons were suddenly eliminated in the system, specific atomic sites that had an additional electron became very unstable, thereby forming bonds with nearby atoms. The formation of the new bonds also induced C–O bond breaking. If bond breakings lead to stable reaction products, those reactions are energetically favored. We speculated that the CO_2_ gas molecule was the most stable among the species generated by available C–O bond breakings.

**Figure 7:**
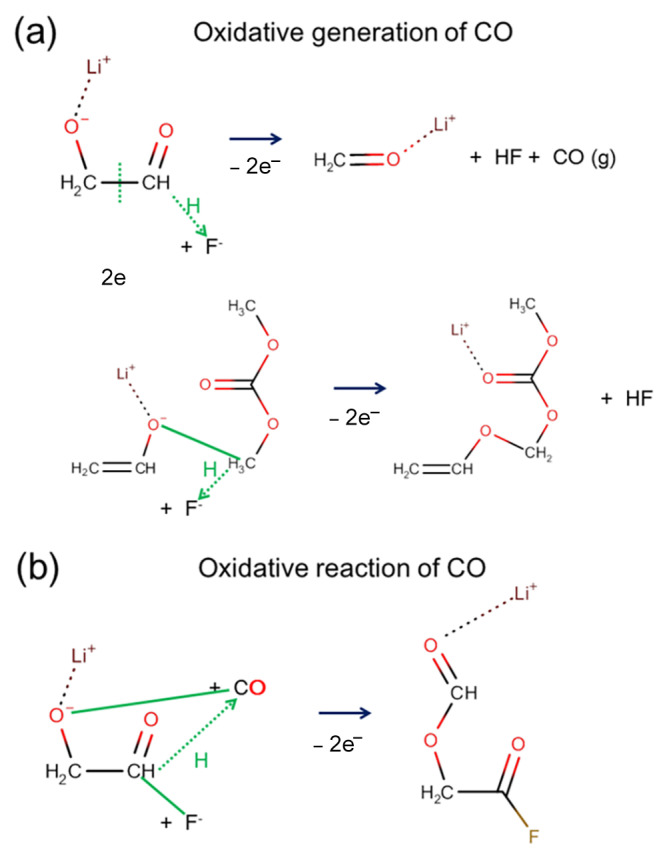
Two reaction pathways show that molecular CO can be either (**a**) generated or (**b**) consumed by the oxidative process. Solid green lines indicate bond formation, and dotted green lines with an arrow indicate hydrogen (proton) transfer.

We also found that the same oxidative stress could occasionally cause CO generation or CO consumption. When two electrons suddenly disappeared, a chemical bond rearrangement occurred, as shown in Figure 7, where the noticeable response involved a proton transfer (marked as a green dotted arrow) and bond formations (marked as solid green lines. The modeling analysis reflects the dynamic characteristics of the gas generation reaction, gas molecules’ generation, and gas molecule consumption that could arise simultaneously depending on the local environment.

## 3. Materials and Methods

### 3.1. Cell Preparation

The main elements of the assembled cylindrical 18650-type cells with a 540 mAh capacity were the following: (i) a cathode, (ii) an anode, and (iii) a separator (Celgard, Charlotte, NC, USA). In this study, the cathode material was based on a slurry mixture of NCM (Ni:Co:Mn = 88:10:2, Ecopro, Ochang-eup, Korea), LITX 200 (Cabot, Billerica, MA, USA), and polyvinylidene difluoride (PVDF, Solvay, Brussels, Belgium) with a weight ratio of 95:2.5:2.5. Two different anode materials were used to study the electrode dependency for the gas generation: (i) a blend of graphite materials (MC09:MC20:MC10 = 45:45:10) and an SBR:CMC (1.5:1) binder with a 97.5:2.5 weight ratio; and (ii) a 94.5:0.5:5 weight ratio mixture of silicon-carbon nanocomposite (SCN), vapor-grown carbon fiber (VGCF), and AG binder. For each cell, 1.6 mL of electrolyte was injected. The reference electrolyte was 1.15 M LiPF_6_ in a solvent mixture of fluoroethylene carbonate (FEC), ethylene carbonate (EC), dimethyl carbonate (DMC), and ethyl methyl carbonate (EMC) with a volumetric ratio of 5:20:35:40. Three additional electrolytes were prepared by adding varying quantities of the divinyl sulfone (DVSF, TCI) additive to the reference electrolyte.

The cylindrical 18650 type of cells were used for electrochemical characterization, and every measurement was performed twice to ensure reliable data acquisition. The chamber temperature was maintained at 25 °C during the electrochemical measurements. The voltage sweep range was 2.8–4.3 V.

For the volumetric measurements of the generated gas as a function of time, three identical battery cells that employed the same electrode and electrolyte composition were manufactured. The gas generation out of each cell was measured at three different points in time: (i) formation cell; (ii) 10 days of storage; and (iii) 30 days of storage. In order to prepare the formation cell, the cells were initially cycled at a rate of 0.2C/0.2C and then cycled again at a rate of 0.5C/0.2C to check capacity. Each of the storage cells was kept in a 60 °C chamber for 10~30 days after fully charging the formation cells.

### 3.2. Gas Measurement

The characteristics of the evolved gas were measured with two methods. One was a quantitative method that used the gas pressure difference before and after accessing the cell. For the closed system, the gas pressure measurement system (Mulimtech, Daejeon, Korea) was reliably disconnected from the air and vented before measurement. After measurement, the conversion of the gas pressure difference to volume was calculated using the ideal gas law. The void volume of this system was carefully minimized as much as possible for reliable measurements.

The other gas measurement method was both qualitative and quantitative. For this measurement, a refinery gas analyzer (RGA, Agilent 7890B, Agilent Technologies, 2015, Santa Clara, CA, USA) was used, and the collected gas from the gas pressure measurement system was analyzed. This RGA system was also used after the vacuum-evacuated process. Each evolved gas component and the amount were analyzed by this method. We found that the sum of each gas component matched extremely well with the gas pressure. To reduce the experimental error, the empty volume of each 18650-type cell was checked at each step and calibrated before the calculations.

The current method of gas measurement was a destructive approach, and measurements showed cumulative amounts at certain points in time. Therefore, repeated or continuous measurement was not available. All of the measured gas amounts were presented after being divided by the weight of the active cathode material.

### 3.3. Computational Methods

In order to investigate the possible mechanisms of reduced gas generation by DVSF additive, we performed ab initio molecular dynamics (AIMD) using the Vienna Ab Initio Simulation Package (VASP) [37,38]. The overall computational scheme proposed in our previous study was adopted here with some variation. To start, the solvation structures were formed in the presence of four Li^+^ excess, and the same number of electrons were added at a point in time for electro-reductive reactions. Since the gas composition analysis in the experiments indicated that DVSF influenced gaseous species mainly from FEC, such as CO and CO_2_, rather than C_2_H_4_ from EC, the only cyclic carbonate we considered in our model was FEC. Therefore, the two compositions that were selected for the model were FEC:DMC:LiPF_6_ (15:10:2) and FEC:DMC:DVSF:LiPF_6_ (15:5:5:2). Note, more cyclic carbonates in computational modeling reflect the electrolyte composition in contact with a polarized surface [39]. If a density of 1.3 g/cm^3^ for the mixture electrolyte is assumed, two formula units of LiPF_6_ in 15.585 and 15.335 Å cubic cells correspond to 0.92 and 0.88 M of salt concentration, respectively.

Since the size of the simulation cell became more tractable compared with what we used previously, we increased the number of structural samplings. To prepare the initial structure for AIMD, we made use of classical molecular dynamics (CMD) with the COMPASS force field [40] as implemented in the Forcite module of Materials Studio. Ten structures were generated using the amorphous cell module [41]. In doing so, each constituting molecule was randomly mixed. With those ten structures, we performed 100 ps of NVT CMD at 500 K. The final structure of an individual run was used as the initial structure for AIMD. Initially, 1 ps of AIMD with a +4 charge state was performed for structural relaxation, and then another 2 ps of AIMD with neutral charge followed to model an electro-reductive reaction. Note that the change of charge state from +4 to neutral (0) equated with an increase in the number of electrons in the electrolyte mixture. Thus, electro-reductive reactions were expected to occur in the following AIMD run. The final structures were analyzed with an in-house tool that examined the bond connectivity between atoms. Gaseous products were easily recognized because our tool printed out each molecular unit with respect to its molecular weight in descending order. A simple summation of gas molecules generated in ten different structures was used to compare the relative quantities of the generated gas. Other than gas generation reactions, other electrochemical reactions were also analyzed in order to determine how DVSF minimized the gaseous products in the electrolyte. After the electro-reductive reactions, we ran another AIMD with four fewer electrons to model the subsequent electro-oxidations. This electro-oxidation was associated with the reactions that occurred when the battery cells were stored. During storage, the battery cells went through spontaneous discharge.

## 4. Conclusions

We experimentally demonstrated that a few percentages of divinyl sulfone (DVSF) additive in liquid electrolyte curtailed the gas generation of a Li-ion battery by more than 50%. In particular, the amount of formation gas turned out to be smaller than that of the cells where the electrolyte without DVSF was used. The differential capacitance data, together with density functional theory (DFT) calculations, show that the π* state of C=C double-bonded carbons in the vinyl group accommodated electrons when additional electrons were added. The outermost carbon atom of DVSF became reactive after receiving the electron, thereby forming a bond with the carbonyl carbon of FEC that was another reactive carbon when FEC received electrons. Once this bond was formed, the reaction pathway from FEC toward gas generation was blocked. In particular, we found that CO_2_ generation from FEC occurred when the electrolyte that had previously undergone a reductive reaction was exposed to the oxidative condition. This reaction pathway for CO_2_ generation was also blocked when electrochemically reduced DVSF formed a bond with a carbonyl carbon of cyclic carbonate. Both the experimental data on the gas-reducing effect of DVSF and the computational study on the mechanism behind it offer valuable insight into the design of new functional additives for battery electrolytes.

## 5. Patents

One US patent relevant to this work is registered with the following ID: US10847841.

## Figures and Tables

**Table 1:** Newly generated gas molecules through reductive reactions and following oxidation reactions of mixture electrolyte with and without the divinyl sulfone (DVSF) additive.

	FEC + DMC				FEC + DMC + DVSF			
	Red		Red + Ox		Red		Red + Ox	
	CO	CO_2_	CO	CO_2_	CO	CO_2_	CO	CO_2_
Str. -01				2				
Str. -02	2		2					
Str. -03	1		1	1				
Str. -04	1		1	1		1		2
Str. -05	1		1	1				
Str. -06	1		2	1				
Str. -07	1		2					
Str. -08	1		1					
Str. -09	2		2					
Str. -10	1		2	1				
Total	11	0	14	7	0	1	0	2

## Data Availability

The data are not publicly available due to corporate policy.

## References

[1] Goodenough J.B., Kim Y. (2010). Challenges for Rechargeable Li Batteries. Chem. Mater..

[2] Etacheri V., Marom R., Elazari R., Salitra G., Aurbach D. (2011). Challenges in the Development of Advanced Li-Ion Batteries: A Review. Energy Environ. Sci..

[3] An S.J., Li J., Daniel C., Mohanty D., Nagpure S., Wood D.L. (2016). The State of Understanding of the Lithium-Ion-Battery Graphite Solid Electrolyte Interphase (SEI) and Its Relationship to Formation Cycling. Carbon.

[4] Aiken C.P., Xia J., Wang D.Y., Stevens D.A., Trussler S., Dahn J.R. (2014). An Apparatus for the Study of In Situ Gas Evolution in Li-Ion Pouch Cells. J. Electrochem. Soc..

[5] Yun K.-S., Pai S.J., Yeo B.C., Lee K.-R., Kim S.-J., Han S.S. (2017). Simulation Protocol for Prediction of a Solid-Electrolyte Interphase on the Silicon-Based Anodes of a Lithium-Ion Battery: ReaxFF Reactive Force Field. J. Phys. Chem. Lett..

[6] Xu K. (2014). Electrolytes and Interphases in Li-Ion Batteries and Beyond. Chem. Rev..

[7] Min K., Seo S.-W., Choi B., Park K., Cho E. (2017). Computational Screening for Design of Optimal Coating Materials to Suppress Gas Evolution in Li-Ion Battery Cathodes. ACS Appl. Mater. Interfaces.

[8] Ushirogata K., Sodeyama K., Okuno Y., Tateyama Y. (2013). Additive Effect on Reductive Decomposition and Binding of Carbonate-Based Solvent toward Solid Electrolyte Interphase Formation in Lithium-Ion Battery. J. Am. Chem. Soc..

[9] Xu K., Angell C.A. (2002). Sulfone-Based Electrolytes for Lithium-Ion Batteries. J. Electrochem. Soc..

[10] Su C.-C., He M., Redfern P.C., Curtiss L.A., Shkrob I.A., Zhang Z. (2017). Oxidatively Stable Fluorinated Sulfone Electrolytes for High Voltage High Energy Lithium-Ion Batteries. Energy Environ. Sci..

[11] Wang Y., Xing L., Li W., Bedrov D. (2013). Why Do Sulfone-Based Electrolytes Show Stability at High Voltages? Insight from Density Functional Theory. J. Phys. Chem. Lett..

[12] Choi N.-S., Han J.-G., Ha S.-Y., Park I., Back C.-K., Lee J., Choi W., Jung I., Lee S., Doo S.G. (2015). Recent Advances in the Electrolytes for Interfacial Stability of High-Voltage Cathodes in Lithium-Ion Batteries. RSC Adv..

[13] Shao N., Sun X.-G., Dai S., Jiang D. (2011). Electrochemical Windows of Sulfone-Based Electrolytes for High-Voltage Li-Ion Batteries. J. Phys. Chem. B.

[14] Leung K., Budzien J.L. (2010). Ab Initio Molecular Dynamics Simulations of the Initial Stages of Solid–Electrolyte Interphase Formation on Lithium Ion Battery Graphitic Anodes. Phys. Chem. Chem. Phys..

[15] Yu J., Balbuena P.B., Budzien J., Leung K. (2011). Hybrid DFT Functional-Based Static and Molecular Dynamics Studies of Excess Electron in Liquid Ethylene Carbonate. J. Electrochem. Soc..

[16] Kim H., Choi W.I., Jang Y., Balasubramanian M., Lee W., Park G.O., Park S.B., Yoo J., Hong J.S., Choi Y.-S. (2018). Exceptional Lithium Storage in a Co(OH)2 Anode: Hydride Formation. ACS Nano.

[17] Kim Y., Um J.H., Lee H., Choi W., Choi W.I., Lee H.S., Kim O.-H., Kim J.M., Cho Y.-H., Yoon W.-S. (2020). Additional Lithium Storage on Dynamic Electrode Surface by Charge Redistribution in Inactive Ru Metal. Small.

[18] Kim H., Son S., Choi W.I., Park G.O., Kim Y., Kim H., Jeong M., Lee H.S., Kim J.M., Yoon W.-S. (2018). Direct Observation of Pseudocapacitive Sodium Storage Behavior in Molybdenum Dioxide Anodes. J. Power Sources.

[19] Choi W.I., Park M.S., Shim Y., Kim D.Y., Kang Y.-S., Lee H.S., Koh M. (2019). Reductive Reactions via Excess Li in Mixture Electrolytes of Li Ion Batteries: An Ab Initio Molecular Dynamics Study. Phys. Chem. Chem. Phys..

[20] Teng X., Zhan C., Bai Y., Ma L., Liu Q., Wu C., Wu F., Yang Y., Lu J., Amine K. (2015). In Situ Analysis of Gas Generation in Lithium-Ion Batteries with Different Carbonate-Based Electrolytes. ACS Appl. Mater. Interfaces.

[21] Choi N.-S., Yew K.H., Lee K.Y., Sung M., Kim H., Kim S.-S. (2006). Effect of Fluoroethylene Carbonate Additive on Interfacial Properties of Silicon Thin-Film Electrode. J. Power Sources.

[22] Shin J.-S., Han C.-H., Jung U.-H., Lee S.-I., Kim H.-J., Kim K. (2002). Effect of Li2CO3 Additive on Gas Generation in Lithium-Ion Batteries. J. Power Sources.

[23] Kim Y. (2013). Investigation of the Gas Evolution in Lithium Ion Batteries: Effect of Free Lithium Compounds in Cathode Materials. J. Solid State Electrochem..

[24] Kim Y. (2013). Mechanism of Gas Evolution from the Cathode of Lithium-Ion Batteries at the Initial Stage of High-Temperature Storage. J. Mater. Sci..

[25] Xia J., Petibon R., Xiao A., Lamanna W.M., Dahn J.R. (2016). Some Fluorinated Carbonates as Electrolyte Additives for Li(Ni0.4Mn0.4Co0.2)O2/Graphite Pouch Cells. J. Electrochem. Soc..

[26] Yoshida H., Fukunaga T., Hazama T., Terasaki M., Mizutani M., Yamachi M. (1997). Degradation Mechanism of Alkyl Carbonate Solvents Used in Lithium-Ion Cells during Initial Charging. J. Power Sources.

[27] Onuki M., Kinoshita S., Sakata Y., Yanagidate M., Otake Y., Ue M., Deguchi M. (2008). Identification of the Source of Evolved Gas in Li-Ion Batteries Using #2#1 -Labeled Solvents. J. Electrochem. Soc..

[28] Kang Y.-S., Park I., Park M.S., Choi W.I., Lee S.Y., Mun J., Choi B., Koh M., Kim D.Y., Park K. (2020). L-Tryptophan: Antioxidant as a Film-Forming Additive for a High-Voltage Cathode. Langmuir.

[29] Kang Y.-S., Yoon T., Mun J., Sik Park M., Song I.-Y., Benayad A., Oh S.M. (2014). Effective Passivation of a High-Voltage Positive Electrode by 5-Hydroxy-1 H -Indazole Additives. J. Mater. Chem. A.

[30] Li Y., Wan S., Veith G.M., Unocic R.R., Paranthaman M.P., Dai S., Sun X.-G. (2017). A Novel Electrolyte Salt Additive for Lithium-Ion Batteries with Voltages Greater than 4.7 V. Adv. Energy Mater..

[31] Wu H.-C., Su C.-Y., Shieh D.-T., Yang M.-H., Wu N.-L. (2006). Enhanced High-Temperature Cycle Life of LiFePO4-Based Li-Ion Batteries by Vinylene Carbonate as Electrolyte Additive. Electrochem. Solid-State Lett..

[32] Zheng J., Engelhard M.H., Mei D., Jiao S., Polzin B.J., Zhang J.-G., Xu W. (2017). Electrolyte Additive Enabled Fast Charging and Stable Cycling Lithium Metal Batteries. Nat. Energy.

[33] Tasaki K. (2005). Solvent Decompositions and Physical Properties of Decomposition Compounds in Li-Ion Battery Electrolytes Studied by DFT Calculations and Molecular Dynamics Simulations. J. Phys. Chem. B.

[34] Shen X., Li P., Liu X., Chen S., Ai X., Yang H., Cao Y. (2021). The Underlying Mechanism for Reduction Stability of Organic Electrolytes in Lithium Secondary Batteries. Chem. Sci..

[35] Han J.-G., Lee J.B., Cha A., Lee T.K., Cho W., Chae S., Kang S.J., Kwak S.K., Cho J., Hong S.Y. (2018). Unsymmetrical Fluorinated Malonatoborate as an Amphoteric Additive for High-Energy-Density Lithium-Ion Batteries. Energy Environ. Sci..

[36] Son H.B., Jeong M.-Y., Han J.-G., Kim K., Kim K.H., Jeong K.-M., Choi N.-S. (2018). Effect of Reductive Cyclic Carbonate Additives and Linear Carbonate Co-Solvents on Fast Chargeability of LiNi_0.6_Co_0.2_Mn_0.2_O_2_/Graphite Cells. J. Power Sources.

[37] Kresse G., Hafner J. (1994). Ab Initio Molecular-Dynamics Simulation of the Liquid-Metal–Amorphous-Semiconductor Transition in Germanium. Phys. Rev. B.

[38] Hafner J. (2008). Ab-Initio Simulations of Materials Using VASP: Density-Functional Theory and Beyond. J. Comput. Chem..

[39] von Wald Cresce A., Borodin O., Xu K. (2012). Correlating Li+ Solvation Sheath Structure with Interphasial Chemistry on Graphite. J. Phys. Chem. C.

[40] Sun H. (1998). COMPASS:  An Ab Initio Force-Field Optimized for Condensed-Phase ApplicationsOverview with Details on Alkane and Benzene Compounds. J. Phys. Chem. B.

[41] Theodorou D.N., Suter U.W. (1986). Atomistic Modeling of Mechanical Properties of Polymeric Glasses. Macromolecules.

